# Relationship between hope and religious beliefs in Turkish women experiencing pregnancy loss

**DOI:** 10.1590/1806-9282.20240792

**Published:** 2025-03-17

**Authors:** Nurdan Kaya Yılmaz, Nazlı Baltacı, Emel Odabaşoğlu

**Affiliations:** 1 Ondokuz Mayıs University, Faculty of Health Sciences, Department of Midwifery – Samsun, Türkiye.; 2 Ondokuz Mayıs University, Faculty of Health Sciences, Department of Nursing, Department of Nursing – Samsun, Türkiye.; 3 Samsun University, Samsun Training and Research Hospital, Child Emergency Clinic – Samsun, Türkiye.

**Keywords:** Hope, Loss, Pregnancy, Religious, Spirituality

## Abstract

**OBJECTIVE:**

The aim of this study was to determine the level of hope and religious attitudes and the relationship between them in women experiencing pregnancy losses.

**METHODS:**

This descriptive and cross-sectional study was conducted on 249 women hospitalized in perinatology clinics. Data from the participants were collected using the "Pregnancy Diagnosis Form," "Dispositional Hope Scale," and "Religious Attitude Scale."

**RESULTS:**

The mean total Religious Attitude Scale and Dispositional Hope Scale scores of the women were 34.02±5.26 and 49.92±8.30, respectively. A significant, albeit weak, positive correlation was observed between the mean total Religious Attitude Scale and Dispositional Hope Scale scores (p<0.01).

**CONCLUSION:**

The Religious Attitude Scale and Dispositional Hope Scale scores of women who experienced a PL were relatively high and above the intermediate level, respectively. Therefore, in women who experienced pregnancy losses, an increase in religious attitudes was associated with an increase in hope for the future.

## INTRODUCTION

Pregnancy loss (PL) refers to the death of an embryo or fetus in the prenatal period. Globally, 2.6 million women experience PLs each year. PL is a major reproductive health problem in both underdeveloped and developing countries^1^. PL is a situational crisis in a woman's life that occurs unexpectedly, inexplicably, suddenly, and traumatically. After the loss, women often experience mental health issues, including anxiety, depression, and posttraumatic stress disorder. Spiritual reactions of women after the loss include resentment toward God, losing the meaning of life, wanting to die, feeling punished, and hopelessness^2^.

Hope is important in coping with the psychological symptoms experienced after a loss and in the healing of the grieving process. Hope improves the mental health of women after a PL and helps them adapt to the grieving process^3,4^. Hope is a type of future-oriented thinking. Emotions reflect the perceived level of hope and, thus, people with high levels of hope are more positive, happier, and optimistic and better at coping with their problems when they achieve their goals. Faith has a great impact on the protection and development of holistic health among women who have experienced PLs in terms of looking to the future with hope^5^. Spirituality, religious beliefs, and faith are precursors that contribute to women's coping with crisis situations and healthy recovery from bereavement. Individuals with strong religious beliefs are known to overcome the crisis and grieving process in a more positive manner^6^.

Respectful maternity care, including bereavement care after a loss, contributes to alleviating the psychological symptoms experienced by the woman and her family. Midwives and nurses are the healthcare professionals who are the first to encounter women who have experienced a loss. After the loss, women are reluctant to talk about their loss because of the associated taboo and stigmatization^7^. Determining the relationship between the level of hope and religious beliefs of women experiencing PLs is believed to make an important contribution to midwifery and nursing science in terms of meeting the care needs of women and their families after a loss.

## METHODS

### Study design and participants

This descriptive and cross-sectional study was conducted on 249 women with PLs at a university hospital in Samsun between July and October 2021. Pregnant women who agreed to participate in the study and were literate were included, whereas those with visual and communication problems were excluded.

### Data measurement

The data of the participating women were collected using the "Pregnancy Diagnosis Form," "Dispositional Hope Scale (DHS)," and "Religious Attitude Scale (RAS)."

- Pregnancy Diagnosis Form: This questionnaire consists of 15 questions to determine some demographic and obstetric characteristics of pregnant women.
- DHS: This scale was developed by Snyder et al.^5^ to determine the level of dispositional hope of individuals aged 15 and above. The validity and reliability study of the scale had been performed in Turkey by Tarhan and Bacanlı^8^. The scale consists of 12 items and two subscales. The total scores that can be obtained from the scale range from 8 to 64, and the scores that can be obtained from the subscales range from 4 to 32. The dispositional hope level of individuals increases with an increase in the scores obtained from the DHS. The Cronbach's alpha coefficient of the scale was 0.86^8^. In our study, the reliability was found to be the same as the original scale..
- RAS: This scale was developed by Ok^9^ to measure individuals’ attitudes toward Islam. This scale consists of eight items and four subscales in total. The total scores that can be obtained from the scale range from 8 to 40. High scores on the scale indicate the presence of high religious attitudes^9^. In this study, the Cronbach's alpha coefficient was 0.89.

### Data collection

The women hospitalized in the perinatology service were informed by the researcher regarding the purpose, content, and importance of the study. Written and verbal consent was obtained from the women. All questionnaire forms were administered in a mean time of 10 min.

### Data analysis

The Statistical Package for Social Sciences (SPSS) 25.0 program was used to analyze the study data. Data were analyzed via independent samples t-test, one-way analysis of variance (ANOVA), Tukey's post hoc test, and Pearson's correlation test. Statistical significance was accepted as p<0.05.

### Ethical considerations

Approval was obtained from the Social and Human Sciences Ethics Committee of Ondokuz Mayıs University (decision date: 28/05/2021, decision no. 2021/464) and from the hospital where the research was conducted. The principles of the Declaration of Helsinki were followed at every stage of the research.

## RESULTS

Of the enrolled women (mean age, 29.71±6.24 years), 46.2% had secondary education, 71.5% were unemployed, 81.1% lived in nuclear families, and 85.1% lived in the city or district centers. According to the results, 79.5% of women had a spontaneous pregnancy and 65.1% of women experienced a PL because of a spontaneous abortion; in addition, 51.0% were in the first trimester. The proportions of women who had planned for and wanted their pregnancies were 65.9 and 78.0%, respectively.

The mean DHS score of the participants was 49.92±8.30, which was above the intermediate level. The study participants had a high level of religious attitude with a mean total RAS score of 34.02±5.26. Average score of the items in the scale (4.25±0.65) indicated that the study participants had a very religious outlook (Table 1).

**Table 1:** The mean Dispositional Hope Scale and Religious Attitude Scale scores and their subscale scores of the participants.

Scales	Subscales	$\bar{\text{X}}\pm \text{SD}$
DHS	Pathway thinking	24.88±4.47
	Agency thinking	25.03±4.51
	Scale total	49.92±8.30
RAS	Cognitive	9.50±1.23
	Behavioral	7.64±2.10
	Emotional	8.14±1.57
	Relational	8.72±1.55
	Scale total	34.02±5.26
	Scale item total	4.25±0.65

$\bar{\text{X}}$ : mean, SD: standard deviation; DHS: Dispositional Hope Scale; RAS: Religious Attitude Scale.

Women experiencing spontaneous abortions and women who wanted their pregnancies had higher mean scores on DHS and the difference was significant (p=0.008 and 0.047, respectively). In addition, the participants’ mean DHS scores appeared to increase with an increase in the number of living children (p=0.023). Participants with a PL in the first trimester, women with a planned pregnancy, and women who wished to have a pregnancy had higher mean RAS scores and the difference was significant (p=0.003, 0.014, and 0.000, respectively). In addition, as the number of pregnancies and the number of living children increased, the mean scores of the RAS increased (p=0.027 and 0.002, respectively) (Table 2).

**Table 2:** Distribution of the mean Dispositional Hope Scale and Religious Attitude Scale scores of women according to some obstetric characteristics.

Characteristics		DHS $\bar{\text{X}}\pm \text{SD}$	RAS $\bar{\text{X}}\pm \text{SD}$
Medical diagnosis	Spontaneous abortions (n=162)	50.83±7.14^a^	34.21±5.57
	Medical abortions (n=40)	46.32±10.93^b^	33.15±5.71
	Stillbirths (n=47)	49.82±8.81^b^	34.08±4.22
	F/p	4.894/**0.008**	0.066/0.517
Gestational age	First trimester (n=127)	50.87±7.49	34.86±5.71^a^
	Second trimester (n=92)	48.54±8.51	31.96±4.62^a^
	Third trimester (n=30)	50.13±10.39	36.46±4.13^b^
	F/p	2.132/0.121	5.886/**0.003**
Pregnancy type	Spontaneous (n=198)	49.90±8.54	34.30±5.33
	Assisted reproductive technique (n=51)	49.98±7.38	32.92±4.86
	t/p	0.055/0.957	−1.678/0.095
Pregnancy planning status	Planned (n=164)	50.62±8.70	34.60±5.48
	Unplanned (n=85)	48.57±7.33	32.88±4.61
	t/p	1.852/0.065	2.482/**0.014**
Intention to have this pregnancy	Wanted (n=194)	50.47±8.54	34.74±5.24
	Unwanted (n=55)	47.96±7.09	31.47±4.50
	t/p	1.995/**0.047**	4.202/**0.000**
Age	r/p	0.025/0.346	−0.053/0.209
Number of pregnancies	r/p	0.040/0.263	0.122/**0.027**
Number of PLs	r/p	−0.087/0.085	−0.024/0.351
Number of living children	r/p	0.126/**0.023**	0.180/**0.002**

PLs: pregnancy losses, t: independent groups t-test, F: one-way analysis of variance, r: Pearson's correlation coefficient; $\bar{\text{X}}$ : mean, SD: standard deviation; DHS: Dispositional Hope Scale; RAS: Religious Attitude Scale.

a,b According to Tukey's test, a significant difference is noted between groups with different letters.

Statistically significant values are denoted in bold.

A significant, though weak, positive correlation was observed between the total and subscale scores of DHS and RAS (p<0.01) (Table 3).

**Table 3:** The relationship between women's mean total scale and subscale scores of Dispositional Hope Scale and Religious Attitude Scale.

DHS and subscales	RAS and subscales				
	Total	Cognitive	Behavioral	Emotional	Relational
Total	r=*0.390*	r=*0.090*	r=*0.410*	r=*0.380*	r=*0.308*
	p=**0.000**	p=*0.156*	p=**0.000**	p=**0.000**	p=**0.000**
Pathway thinking	r=*0.411*	r=*0.089*	r=*0.422*	r=*0.383*	r=*0.361*
	p=**0.000**	p=*0.160*	p=**0.000**	p=**0.000**	p=**0.000**
Agency thinking	r=*0.309*	r=*0.077*	r=*0.335*	r=*0.319*	r=*0.209*
	p=**0.000**	p=*0.225*	p=**0.000**	p=**0.000**	p=**0.001**

r: Pearson's correlation coefficient; DHS: Dispositional Hope Scale; RAS: Religious Attitude Scale. Statistically significant values are denoted in bold and italic.

## DISCUSSION

Hope sense is important for all women after a loss. Some women who experience grief after a loss experience a loss of hope; however, for others, hope is a motivating force in planning their life and future^4^. In a study conducted on women after a PL for the first time, it was found that they experienced emotions changing between hope and hopelessness after the loss^10^. In the present study, the hope levels of women appeared to be above the intermediate level. In the literature, studies investigating the hope levels of women after a loss are limited. However, in these studies, women's hope levels were intermediate^11,12^. Therefore, the hope levels of all women after a loss were similar.

Hope helps alleviate the physical and psychological discomfort following spontaneous abortions. A study in Iran reported that hope therapy improved the quality of life and psychological well-being of women after abortions^13^. In this study, the DHS levels of women who had spontaneous abortions were higher than those of women who had medical abortions and stillbirths. Spontaneous abortion is one of the most common pregnancy complications^1^. Therefore, women accept that spontaneous abortions are more common than other causes of perinatal loss, and in this context, their hope levels are higher.

In pregnancy, the woman begins to step into the role of motherhood, dreams about her future baby, and waits for the future with hope. However, not all women react and adapt to and accept pregnancy in the same way. While women who conceive intentionally have higher levels of pregnancy adjustment^14^, women who experience an unintended pregnancy are more likely to experience psychological distress^15^. In this study, hope levels were higher in those who became pregnant voluntarily. A study reported that individuals with children had higher levels of dispositional hope than individuals without^16^. Hope in human life is knowing the ways to achieve the future one desires and motivating oneself to implement these ways^5^. In this context, the high level of hope in women who have children is thought to be attributable to the fact that they have experienced successful pregnancy and childbirth in the past.

Religious beliefs and attitudes help women cope with grief after PLs^6^. In a study conducted on Polish women, a positive and significant relationship was observed between life satisfaction and religious beliefs after stillbirths^2^. In their study, women had a high religious view with a high level of religious attitude. A study in Turkey found that women with risky pregnancies had a mean RAS score^17^ similar to this study. The fact that all women included in their study were Muslim may explain this similarity.

Losses occurring up to the 20th week of pregnancies are defined as miscarriages and after the 20th week are described as stillbirths^1^. The present study found that women's religious attitudes were notably higher in the first trimester. In a study conducted on women experiencing early, middle, and late PLs, it was reported that religious belief is important in improving the grief process after a loss in all periods^18^. Another study stated that 72.7% of women who had a spontaneous abortion had higher levels of religious attitudes than those who had other types of abortions^3^. Spontaneous abortions mostly occur in the first trimester and are more common than other types of losses. In this context, the higher religious attitudes of women after a loss in the first trimester in the present study spontaneous abortions were four times more common than medical abortions and may have resulted from the fact that stillbirths.

In the present study shows that as the religious beliefs of women who have experienced pregnancy loss increase, their levels of hope also rise. In a study conducted in China on women receiving chemotherapy after a diagnosis of breast cancer, those with high levels of religious attitudes had high levels of hope^19^. Similarly, a study conducted in India on women diagnosed with breast cancer reported a positive relationship between spirituality and hope^12^. Therefore, religious elements in women appear to be associated with having hope, which can be very effective in coping, and that they can be handled together in the care process. Based on all this information, it is necessary to establish combined management protocols for pregnant women that include spiritual health, which includes elements related to religion and hope as well as psychological health, and to operate in an integrated manner in the care process.

The limitation of this study is owing to its single-centered nature, the results cannot be generalized to all women.

## CONCLUSION

This study revealed that women after PLs had high levels of religious attitudes and hope levels above the intermediate level. Women who had spontaneous abortions and wanted their pregnancies had higher levels of DHS. Women's dispositional hope levels increased as the number of living children increased. Women after a loss in the first trimester and women whose pregnancies were planned and voluntary had relatively high religious attitudes. Their religious attitudes increased with an increase in the number of pregnancies and the number of living children. As the religious attitudes of women who have experienced a PL increased, their level of hope increased.

According to the findings of the study, psychosocial and spiritual care procedures should be designed and implemented in the hospital and at home after discharge to improve women's spiritual, psychological, and emotional health following PLs. Furthermore, the spiritual needs of pregnant women can be met by providing information and counseling about pregnancy, birth, and postnatal processes; identifying and monitoring the spiritual and social support needs of pregnant women; and creating a care environment suitable for these needs.
